# Measurement of the cleavage energy of graphite

**DOI:** 10.1038/ncomms8853

**Published:** 2015-08-28

**Authors:** Wen Wang, Shuyang Dai, Xide Li, Jiarui Yang, David J. Srolovitz, Quanshui Zheng

**Affiliations:** 1 Department of Engineering Mechanics, Center for Nano and Micro Mechanics, Applied Mechanics Lab, Tsinghua University, Beijing 100084, China; 2 Department of Materials Science and Engineering, University of Pennsylvania, Philadelphia, Pennsylvania 19104, USA; 3 Department of Mechanical Engineering and Applied Mechanics, University of Pennsylvania, Philadelphia, Pennsylvania 19104, USA; 4 XIN Center, Tsinghua University, Beijing 100084, China; 5 State Key Laboratory of Tribology, Tsinghua University, Beijing 100084, China

## Abstract

The basal plane cleavage energy (CE) of graphite is a key material parameter for understanding many of the unusual properties of graphite, graphene and carbon nanotubes. Nonetheless, a wide range of values for the CE has been reported and no consensus has yet emerged. Here we report the first direct, accurate experimental measurement of the CE of graphite using a novel method based on the self-retraction phenomenon in graphite. The measured value, 0.37±0.01 J m^−2^ for the incommensurate state of bicrystal graphite, is nearly invariant with respect to temperature (22 °C≤*T*≤198 °C) and bicrystal twist angle, and insensitive to impurities from the atmosphere. The CE for the ideal ABAB graphite stacking, 0.39±0.02 J m^−2^, is calculated based on a combination of the measured CE and a theoretical calculation. These experimental measurements are also ideal for use in evaluating the efficacy of competing theoretical approaches.

## Abstract (web summary)

Knowing the cleavage energy of graphite layers is important to understand the mechanical properties of graphite, graphene and related compounds. Here, the authors report a high accuracy experimental measurement of the cleavage energy and validation through multiscale modelling.

## Graphical abstract

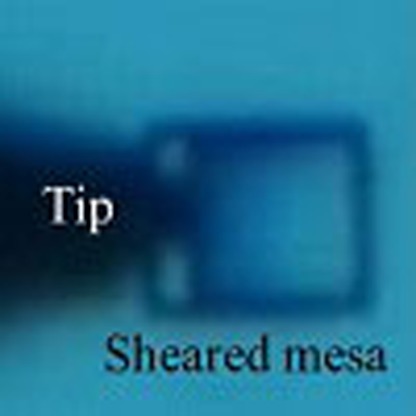

Graphite is the most stable form of carbon under standard conditions and is a layered, hexagonal (*P*6_3_/*mmc*) crystal. Each layer is a one-atom thick graphene sheet, in which carbon atoms are arranged in a two-dimensional (2D) honeycomb lattice (space–plane groups *P*6/*mmc*–*p*6*mm*)^1^,^2^,^3^. Compared with the extremely strong *sp*^2^ intralayer bonds, the interlayer interactions are controlled by much weaker van der Waals bonding. This contrast leads to many novel physical and mechanical properties of graphite, such as maximal values of the electric and thermal conductivities, in-plane elastic stiffness and strength^2^,^4^,^5^,^6^,^7^,^8^,^9^, and the minimum shear-to-tensile stiffness ratio^10^. These novel properties make graphite, graphene and their allotropes (carbon nanotubes and fullerenes) of intense interest for a wide range of applications.

In spite of a very large and rapidly growing literature on graphite, graphene and their allotropes, a quantitative understanding and characterization of the interlayer interactions of graphite has yet to emerge^11^,^12^,^13^,^14^,^15^,^16^,^17^,^18^,^19^,^20^. The interlayer binding energy is a relatively simple measure of the interlayer interactions and is defined as the energy per layer per area required separating graphite into individual graphene sheets (for example, by uniformly expanding the lattice in the direction perpendicular to the basal plane). This energy is nearly equivalent to the exfoliation energy and is approximately equal to the cleavage energy (CE, the energy to separate a crystal into two parts along a basal plane) and twice the basal plane surface energy. On the theoretical side, direct calculation based on conventional density functionals cannot correctly represents the long-range van der Waals nature of interlayer interactions in graphite^21^. Recently, several approaches have been suggested to overcome this deficiency, such as Grimme's density functional correction^22^, a non-local functional^23^, the meta-generalized gradient approximation^24^,^25^, the adiabatic-connection fluctuation-dissipation theorem within the random phase approximation (ACFDT-RPA)^26^ and quantum Monte Carlo (QMC) calculations^27^,^28^. From an experimental perspective, the situation is also murky; there are no reliable, direct measurements of these energies in graphite; previous indirect measurement approaches yield values that range from 0.14 to 0.72 J m^−2^ (see Supplementary Table 1 and Supplementary Note 1 for a summary) and no consensus has emerged. Here, we report the first direct and accurate experimental measurement of the CE of graphite. The method we adopted is based on the recently discovered self-retraction phenomenon in graphite^29^.

## Results

### Experimental methods of measuring the CE

**Figure 1:**
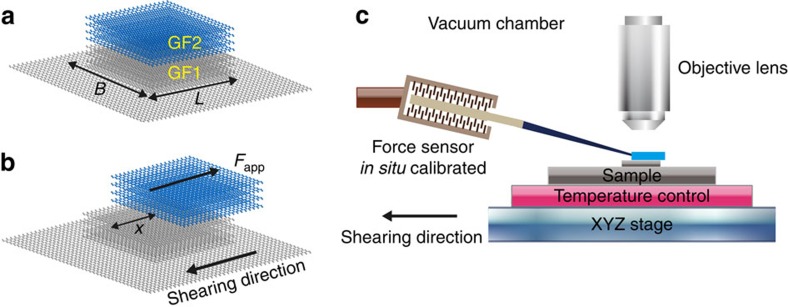
Illustration of the cleavage energy measurement. (**a**) The graphite sample is a stack of two thin, single crystal, rectangular graphite flakes, GF1 (blue) and GF2 (grey), with parallel basal planes, but rotated with respect to each other about [0001] by an angle, *φ*. Hence, the (0001) planar interface between GF1 and GF2 is a twist grain boundary on the (0001) plane. The sample is fixed to a rigid substrate. (**b**) The cleavage energy is measured through shearing the lower flake relative to the upper one in the superlubric state. (**c**) Schematic illustration of the experimental setup to shear the sample using an XYZ stage, measure the shear force, *F*_app_, using an *in situ* calibrated (see Methods) and fixed micro-force sensor, and control the temperature and vacuum.

Our experimental method for measuring the CE can be better understood in terms of an ideal experiment performed in absolute vacuum as described below. The sample is a rectangular graphite plate fixed to a rigid substrate. The plate itself is a stack of two thinner, single crystal, rectangular graphite flakes GF1 and GF2 (grey and blue flakes in Fig. 1a), with the (0001) basal planes of both flakes parallel, as illustrated in Fig. 1a. The orientations of the two single crystal flakes are not the same, but are rotated with respect to one another by an angle, *φ* (0°<*φ*<60°) about the [0001] direction. The interface between GF1 and GF2 is a twist grain boundary lying on a (0001) plane with an energy per unit contact area, *σ*(*φ*). The CE of these two flakes is thus *Γ*_0001_(*φ*)=2*γ*_0001_−*σ*(*φ*), where *γ*_0001_ represents the (0001) surface energy of graphite (of course, at *φ*=0, *σ*=0 and *Γ*_0001_=2*γ*_0001_). For simplicity, we drop the 0001 subscript since that the remainder paper refers only to the basal plane of graphite.

Recent experimental observations showed that the contact between two rotated single crystal basal-oriented graphite flakes is superlubric, namely, the contact is (nearly) frictionless^30^,^31^,^32^,^33^. Thus, when slowly shearing a distance *x* (Fig. 1b), two new free (0001) surfaces with total area 2*Bx* are exposed, where *B* denotes the flake width. The total free energy changes by *G*=(2*γ*−*σ*(*φ*))*Bx*=*Γ*(*φ*)*Bx*>0. As a consequence, a driving force, *F*_ret_=−(d*G*)/(d*x*)=−*Γ*(*φ*)*B* (neglecting any dissipation that may occur—see below), exists for the flake to retract back to its original position (Fig. 1a) in order to reduce the free energy. Therefore, in the superlubric state, the cleavage energy *Γ*(*φ*) can be determined through a precise measurement of the applied shear force, *F*_app_, required to balance the retraction force *F*_ret_ in the quasi-static loading (shearing) and unloading (retraction) processes: *Γ*(*φ*)=*F*_app_/*B*. In addition, we note that precise measure of the width *B* is straightforward, and that the superlubric retraction process was only recently observed^29^.

To perform these experiment, graphite mesas were prepared using the technique reported in refs 29, 31 with the same highly ordered pyrolytic graphite, HOPG (Veeco ZYH and ZYB grade). The HOPG has a brick wall-like polycrystal structure^31^ in which each grain is from a few to tens of micrometres wide (parallel to the basal plane) and three orders of magnitude smaller in the perpendicular [0001] direction, ranging from a few to tens of nanometres^31^,^34^. The grains are stacked such that they share a common [0001] direction but are randomly oriented with respect to that axis. This implies that the [0001] interfaces are planer and pure twist. For our measurements, we prepare mesas with edge lengths 2≤*B*≤9 μm and heights of ∼1 μm. Given the dimensions of the grains, mesas frequently have at least one grain boundary parallel to the free surface that runs across the entire mesa^31^; the grain boundary is the interface between the upper graphite flake (GF2 blue) and the lower graphite flake (GF1 grey) associated with a rotation of GF2 relative to GF1 about a common [0001] axis, as indicated in Fig. 1a,b. These twist grain boundaries (interface between GF2 and GF1) are superlubric contacts.

The experimental setup is schematically illustrated in Fig. 1c: a micro-force sensing probe (FemtoTools FT-S100 with a 5 nN force resolution and a bandwidth of up to 8 kHz) is fixed to a micro-manipulator (Kleindiek MM3A); the temperature and applied shear force were controlled by placing the test samples on a ceramic heating plate affixed to a XYZ stage (XMT XP-611) that can be translated in three dimensions with high precision. The force measurements were made while the upper flake GF1 was stationary with the force sensor probe tip pushing on the side edge of the upper flake GF1 in order to provide a lateral force that shears the flake (the probe and sample are simply pushed into contact). The possibility of an oblique sensor/GF1 contact is unimportant because the sensor is calibrated *in situ* for each measurement (see Methods for details). In our measurements, the typical rates at which graphite flake GF1 was translated was ∼25 nm s^−1^. All of the measurements were performed under an optical microscope (Carl Zeiss Axio Scope.A1).

### Analysis of the measured curves

**Figure 2:**
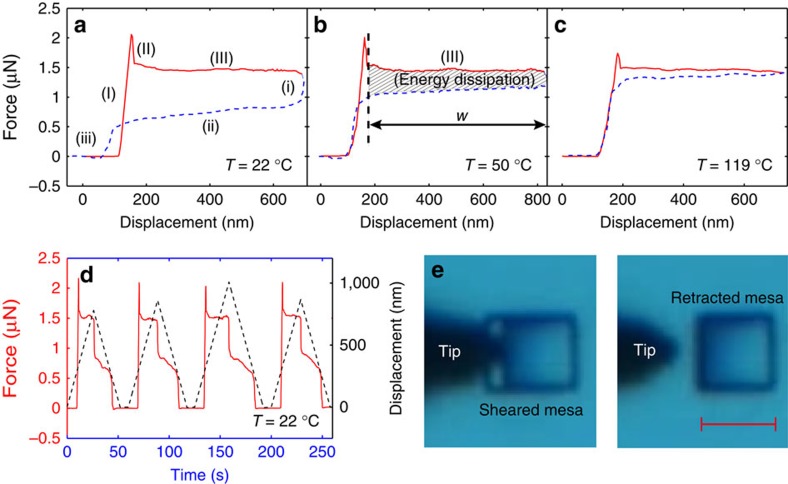
Measured force–shear displacement curves. Typical measured force–shear displacement curves for loading (red solid line) and unloading (blue dashed line) in ambient conditions at temperatures (**a**) 22 °C, (**b**) 50 °C and (**c**) 119 °C. The shaded area in **b** between the entire region (III) loading curve and the unloading curve is the energy dissipated in sliding and retraction. This energy dissipation can be normalized by the region (III) width, *w*, and the sample width *B* to find the dissipative energy (see more below). (**d**) The time traces of the shearing distance (black dashed line) and the shear force (red solid line) at 22 °C. (**e**) Optical images of the sheared mesa (left) and retracted mesa (right). Scale bar, 4 μm.

We first tested several graphite mesas to verify that self-retraction occurs. For such mesas, we measured the forces and shear displacements of the top flake both during loading and unloading. Figure 2a shows a typical force–displacement curve for loading and unloading under ambient conditions (temperature 22±1 °C, relative humidity 25±2%) for a square mesa with side length *B*=4 μm and height *H*=1 μm. The loading curve can be divided into three regions: (I) a nearly linear shear force–displacement region which characterizes the predominantly elastic deformation of the tip before the applied force exceeds the sum of the retraction and static friction force; (II) a sudden drop of the shear force which corresponds to breaking the chemical bonds at the sample edges formed during the reactive ion etch used in fabricating the mesas; and (III) a nearly constant shear force where the applied force *F*_app_ balances the retraction force *F*_ret_, *F*_app_=−*F*_ret_ in the superlubric state, where friction is negligible. The slope is zero in loading region III since the advancing flake creates new, contaminant-free surfaces as it moves. Figure 2d shows typical time traces of the shearing distance (black dashed line) *x* and the measured shear force (red solid line) *F*_app_ for the same mesa. We observed nearly the same force plateaus in the loading and unloading over at least 10 cycles. The results are robust to changes in sample mesas tested and temperatures, clearly showing the excellent repeatability of the measurements.

Since the loading and unloading cycle required ∼100 s, the exposed surfaces can adsorb a significant quantity of contaminants under ambient conditions^35^. During loading, the advancing flake creates new, contaminant-free surfaces. However, the retracting flake slides over existing surfaces on which there are contaminants. The retracting flake tends to sweep these contaminants^36^ ahead of the flake edge in a push broom-like motion that dissipates energy leading to a contamination (or cleaning) friction *F*_cf_. This contamination friction force is proportional to the edge (that is, the edge of GF2 in contact with the free surface of GF1) length. The unloading curve can also be divided into three regions: (i) an elastic unloading of the tip until *F*_app_≤–(*F*_cf_+*F*_ret_) (recall that *F*_ret_<0 and *F*_cf_>0); (ii) a region where *F*_cf_ increases with retraction distance (the advancing flake edge pushes contaminants ahead of itself—the quantity of contaminant pushed grows in proportion to the flake retraction distance); and (iii) a rapidly decreasing force where GF2 returns to its original position—this overlaps the initial loading region (I) reflecting the elastic unloading of the tip after the upper flake returns to its initial position.

**Figure 3:**
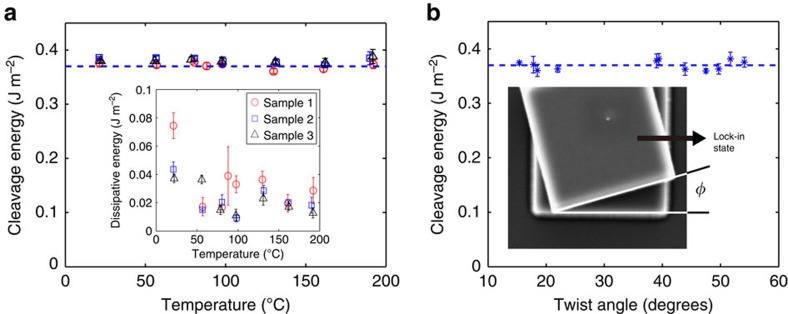
Temperature and twist angle dependence of the CE. (**a**) The measured cleavage energy as a function of temperature. Red, blue and black symbols represent three different samples with the same side length (*B*=4 μm) and the error bars represent the s.d. of five independent measurements on each. The inset shows the dissipative energy, defined in the caption of Fig. 2b, versus temperatures. (**b**) The measured cleavage energy as a function of twist angle *φ* at *T*=20 °C. The CE values were measured from 11 different samples with the same side length (*B*=3 μm). Each sample corresponds to a different twist angle. The error bars represent the standard deviation of five independent measurements for each sample. The insert shows a typical sample at lock-in from which the rotation angle is measured.

To validate the conjectured role of impurities in creating *F*_cf_, we performed similar loading and unloading measurements for the same mesa as a function of temperature in the same environment (Fig. 2a–c). The expectation is that increasing temperature reduces the equilibrium impurity concentration on the newly exposed surfaces^35^. Examination of Fig. 2a–c shows that the gap between the loading and unloading curves and the slope on the unloading (retraction) curve (region (ii)) decreases with increasing temperature. The decrease in the slope in the *F*_app_ versus displacement curve in unloading (region (ii)) with increasing temperature is associated with decreased impurity concentration on the surface at higher temperature; recall that *F*_cf_ is proportional to the area of the surface swept (sliding distance) during translation of the upper crystal with respect to the lower one during retraction. Hence, *F*_cf_ should go to zero in the high temperature limit (see Fig. 3a inset); the temperature at which this term becomes negligible should scale in proportion to the contaminant—surface binding energy. The fact that the loading and unloading curves are nearly identical at the highest temperature (119 °C) demonstrates that there is little hysteresis in the sliding/retraction process. Additional results over a wider temperature range are shown in Fig. 3a. Additionally, the fact that the loading curve in region (III) is nearly identical to the unloading (retraction) curve in region (ii) at slightly elevated temperatures (Fig. 2c) demonstrates that the magnitude of the dynamic friction force is negligible (since this force points in opposite directions on loading and unloading) and the retraction (above ∼100 °C) is superlubric. Finally, we note that since the loading curve is flat and temperature independent, the flake translation on loading is superlubric over the entire temperature range examined.

These observations, taken together, clearly demonstrate that *F*_app_=–*F*_ret_=*BΓ*(*φ*) or that measurements of *F*_app_ (in region (III)) and the sample width (*B*) give the CE, *Γ*(*φ*)=*F*_app_/*B*. In this manner, plus the precise measurement of the mesa width *B* using a scanning electron microsopy (Quanta FEG 450), we find an average CE of *Γ*(*φ*)=0.37±0.01 J m^−2^, where the data was averaged over 50 samples with 2–9 μm flakes with rotation angles 16°≤*φ*≤54°.

### Temperature and twist angle dependence of the CE

At finite temperature, we expect the individual basal planes to fluctuate. This could give rise to a thermal effect on the CE; such an effect has not heretofore been reported. We experimentally investigate the impact of temperature in ambient laboratory conditions (at a relative humidity of 22±5% and with different temperatures from 22 to 198 °C). Figure 3a shows the measured CE as a function of temperature (based on the loading curves). From these results, we see that the CE of incommensurate (large twist angle) graphite is nearly temperature invariant over the temperature range examined. On the other hand, the shaded area between regions (III) on loading and region (ii) (Fig. 2) is clearly temperature dependent. Normalizing by the force plateau width *w* and the sample length *B* gives an intrinsic measure of this effect. The inset in Fig. 3a shows that the dissipative energy decreases with increasing temperature. As discussed above, this is likely due to decreased contaminant concentration on the surface with increasing temperature. We have not examined whether this represents the equilibrium adsorption isotherm or kinetics plays a role here.

As discussed above, the cleavage energy of graphite *Γ* is the difference between twice the surface energy 2*γ* and the twist grain boundary energy *σ*. Since *σ* is expected to be a function of twist angle *φ* (like grain boundaries in most crystalline materials), so too is *Γ*. The first step in determining this *φ* dependence is the measurement of *φ*. We do this based on the lock-in effect^31^; this refers to the observation that self-retraction disappears at a particular rotation angles of GF2 relative to GF1 (ref. 31). This can be understood as follows: if two crystals have an arbitrary rotation with respect to one another such that they are incommensurate and the two crystals are rigid, there is no barrier to sliding^37^,^38^,^39^. However, when two graphite flakes are commensurate (perfect ABAB stacking) at *φ*=0, the barrier to sliding is the theoretical shear strength of the material. This was observed in ref. 31. By measuring the angle required to rotate GF2 into such a no-retraction condition, we determine the initial rotation of GF2 relative to GF1, that is, *φ*. Figure 3b shows the cleavage energy as a function of twist angle *φ* (the inset shows a typical observation of a flake rotated into the no-retraction condition). These results, obtained from 11 samples of the same side length *B*=3 μm, show that over the range of angles examined (16°≤*φ*≤54°), the cleavage energy is surprisingly independent of twist angle *φ*.

### Theoretical calculations

While several measurements and predictions (Supplementary Tables 1 and 2 and Supplementary Note 1) are available for interlayer bonding and the surface energy of graphite (*γ*=*Γ*(0)/2), little information is available on the twist boundary energy *σ*(*φ*). We turn to theoretical analysis to understand both the magnitude of *σ* and its independence on twist angle, *φ*. We do this in the framework of the Peierls–Nabarro model^40^,^41^,^42^ (that was originally formulated to describe dislocations), generalized to account for anisotropic elasticity^43^ and extended to describe twist boundaries^44^,^45^. In this model, the total energy consists of two parts: the elastic energy stored in the crystals on either side of the boundary and the misfit energy that represents the atomic interactions (bonding) between the two crystals (at the grain boundary). The only inputs to the model are the anisotropic elastic constants for graphite and the generalized stacking fault energy (GSFE). The GSFE is simply the total energy of a pair of semi-infinite rigid graphite crystals meeting at a (0001) plane as a function of the shift of the two crystals parallel to that plane minus the energy when the shifts are zero (that is, perfect ABAB stacking). The form of the 2D GSFE function (displacements in two orthogonal directions in the (0001) plane) must respect the symmetry of the graphite crystal structure^44^,^45^. In the Methods part, we describe how the GSFE is obtained based on accurate first-principles calculations and provide all of the functions and parameters used as input to the anisotropic Peierls–Nabarro grain boundary (APNGB) model applied to (0001) twist grain boundaries in graphite.

**Figure 4:**
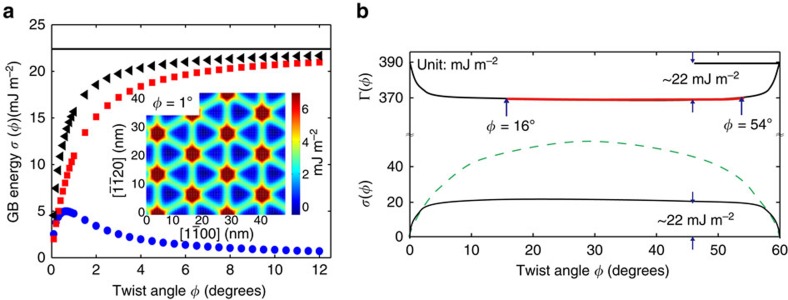
Theoretical results. (**a**) The (0001) twist grain boundary (GB) energy in graphite obtained from the APNGB model as a function of twist angle *φ* (black symbols). The contributions to the GB energy from the elastic and misfit (GSFE) energies are shown by blue and red symbols. The solid black horizontal line shows the GB energy assuming that the graphite crystals are rigid and incommensurate with each other (obtained as the average over the entire GSFE). The inset shows the spatial distribution of the misfit energy in a *φ*=1° twist grain boundary (dislocations cores are seen as green lines). (**b**) The results from **a** plotted over the entire twist angle range (lower black curve) and the corresponding cleavage energy *Γ*(*φ*) over the same range based upon the measured values of *Γ*(*φ*) (shown as the red line). The green dashed curve shown for comparison, is the typical shape of *σ*(*φ*) for a twist GB on the (111) plane of a fcc metal^45^.

For small, twist angles *φ*, the grain boundary can be thought of as a 2D array of dislocations^42^,^44^, as shown in the inset to Fig. 4a for a 1° twist angle from the APNGB calculation. The green lines (intermediate misfit energy) in that figure represent the dislocation cores and the red regions with highest misfit energy are the positions where dislocation lines intersect. These results show that the dislocation core width is *w*≃3 nm (the width of the green lines in the inset to Fig. 4a). This exceptionally large dislocation core width is associated with the very weak interlayer bonding and relatively strong/stiff intralayer bonding in graphite and is consistent with electron microscopy observations^46^. The nearly triangular dislocation array is associated with the dissociation of the screw dislocations in this boundary into partial dislocations and alternating triangles correspond to regions of ABAB|ABAB (perfect crystal) stacking and ABAB|CACA stacking (that is, a stacking fault). The magnitude of the stacking fault energy in graphite is very small, ∼0.85 mJ m^−2^ (see Methods). The corresponding twist boundary energy versus twist angle is shown in Fig. 4a. The energy rises rapidly from zero at 0° and saturates at ∼22 mJ m^−2^ over a characteristic angle range of 4° (90% of the saturation value). The saturation of the twist boundary energy at such a small angle is unusual compared with non-van der Waals bonded materials (for example, metals^44^,^45^) and can be understood as the angle for which the dislocation cores overlap. The dislocation spacing is *d*≃*b*/*φ*, where *b* is the magnitude of the Burgers vector (∼0.2 nm for partial dislocations in graphite). Hence, the critical angle for dislocation overlap, that is, *d*≃*w*, is 
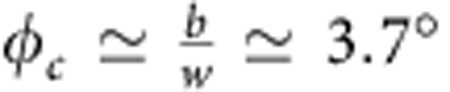
, in good agreement with the APNGB results. A similar condition applies at 60°–*φ*_c_, where the 60° rotation corresponds to a perfect twin with extremely small energy. For twist angles in the range *φ*_c_≤*φ*≤60°−*φ*_c_, the dislocation cores significantly overlap and the twist boundary can be viewed as two rigid crystals meeting incommensurately at the twist boundary. The energy of such a configuration is almost entirely the result of the misfit energy (the elastic energy is negligible over this angle range—Fig. 3a) and can be simply obtained by performing an average over the entire generalized stacking fault energy (see Methods). This is the asymptotic, large-angle grain boundary energy, which is *σ*_0_≃22 mJ m^−2^ for graphite.

These theoretical results can be used to interpret the experimental findings. The cleavage energy is predicted to be nearly independent of twist angle over the entire experimental range from 16° to 54°. This is consistent with the experimental observations (Fig. 3b). The theoretical results show that a variation with twist angle should only be seen for 0°<*φ*<4° or 56°<*φ*<60°. Since the contribution to the cleavage energy from the surface energy is so much larger than the grain boundary energy (and its variation), even for these angles, the variation in *Γ* with *φ* will be small. We can use the theoretical results to estimate the (0001) surface energy from the experimentally measured cleavage energies. Over the experimentally accessible twist angle range, with a measured value of *Γ*=0.37±0.01 J m^−2^, the ideal cleavage energy is *Γ*(0)=*Γ*+*σ*_0_=2*γ*=0.37+0.02 J m^−2^=0.39±0.02 J m^−2^, where *σ*_0_ is the large angle (4°≤*φ*≤56°) value of the twist grain boundary energy. We estimate the error in *σ*_0_ to be less than ∼0.005 J m^−2^ (see Methods).

## Discussion

Graphite is an unusual material; it has very strong (covalent) bonding within the basal plane but has extremely weak (van der Waals) bonding between basal planes. This results in very large (small) values of the elastic constants with components without (with) components in the direction normal to the basal plane. While unusual compared with most materials, it is also prototypical of layered van der Waals bonded systems. Hence, definitive values for the main energetics of this system are both interesting and important. This has led to a wide range of measurements and theoretical predictions of the strength of this bonding (especially as it relates to the interlayer bonding). This interlayer binding energy has been reported in several forms for graphite^47^; namely the CE, the (0001) surface energy, the binding energy and the exfoliation energy (EE, the energy per area required to remove one (0001) atomic layer from the surface of the bulk material). Experimental measurements suggest cleavage energies in the 0.19–0.72 J m^−2^ range (or 0.43±0.29 J m^−2^) and the theoretical predictions are in the 0.03–0.51 J m^−2^ range (or 0.27±0.24 J m^−2^); see Supplementary Tables 1 and 2. Before the present work, direct, accurate experimental measurements of these energies were unavailable and theoretical predictions were routinely confounded by the difficulty of fully including dispersion forces within first-principles frameworks (even the most accurate methods show significant variations). The different measurements of the interlayer bonding are inter-related by either exact relations or by theoretical estimates; surface energy=CE/2, binding energy≈0.85 CE and CE≈0.85 exfoliation energy^47^.

In the present work, we report accurate experimental results for the CE of incommensurate graphite on the basal plane, that is, CE=0.37±0.01 J m^−2^. In order to relate this to the CE of a perfectly stacked AB graphite crystal, we performed APNGB energy calculations based on a combination of experimental and first principle results to obtain a grain boundary energy with a maximum value of *σ*=0.02±0.005 J m^−2^. This implies CE=0.39±0.02 J m^−2^ for perfectly stacked AB graphite. While this value is a combination of experimental and computational results, the uncertainties are still very small and this value should be considered definitive. Using the relations described above, these results imply a basal plane surface energy of 0.20 J m^−2^, an interlayer binding energy of 0.33 J m^−2^ and exfoliation energy of 0.46 J m^−2^. These results provide an excellent means to distinguish between competing approaches for *ab initio* prediction of bonding in van der Waals materials.

## Methods

### Calibration of the micro-force sensing probe

The micro-force sensing probe senses force along its axial direction. For the measurements discussed in the paper, we focus on the force component along the shearing direction (Fig. 1a) *F*_app_=*F*cos*θ*, where *F* is the force along the axial direction and *θ* is the angle between the axis of the micro-force sensing probe and the shearing direction. The accuracy of the measurement depends upon the *in situ* calibration of the micro-force sensor; the calibration was performed using a diamagnetic lateral force calibrator (D-LFC)^48^ in which a square sheet of pyrolytic graphite is levitated above four permanent magnets. The lateral displacement *x* of the pyrolytic graphite is proportional to the force *F* acting upon it, that is, *F*=*kx*, where *k* is the D-LFC spring constant which can be evaluated from *k*=*mω*_n_^2^, and *m* and *ω*_n_ are the mass and natural frequency of the pyrolytic graphite. The natural frequency *ω*_n_ was measured by tracing the trajectory of the pyrolytic graphite using a high speed camera (FASTCAM SA3, PHOTRON, USA). The blue curve in Supplementary Fig. 1a shows a typical measurement of the displacement (in unit of camera pixels) of the pyrolytic graphite sheet as a function of time and the red curve depicts its envelope based on free vibration theory. These two curves are in excellent agreement. Supplementary Figure 1b shows the fast Fourier transform of the displacement–time profile (blue curve in Supplementary Fig. 1a), from which we obtain the natural frequency, that is, *f*=7.2 Hz. Using this frequency, we obtain the D-LFC spring constant, *k*=0.013±0.0002 N m^−1^. This D-LFC spring constant *k* is used to calibrate the micro-force sensor. After each graphite cleavage energy measurement, we replace the graphite sample with the D-LFC to recheck the calibration. During the loading calibration, the D-LFC moved towards the tip of micro-force sensor at constant velocity, *v*. The force sensor produces no output until it contacts the D-LFC. At this point, we start to record both the loading time (*t*) and the force sensor voltage output (*S*). The sensitivity of the force sensor, *kvt*/*S*, determined in this way, is 0.659±0.002 μN V^−1^. Supplementary Figure 1c,d shows the loading and unloading calibration results for the micro-force sensing probe. The blue line represents the force output of the D-LFC, the red dots represent the voltage output of the force sensor before calibration and the blue dotted line represents the force output of the force sensor after calibration.

### Theoretical method

In the paper, we report the (0001) twist grain boundary energy *σ* as a function of the twist angle *φ* for 0.1°≤*φ*≤12° based on an APNGB model, as described in detail in refs 43, 44. This model had previously been applied to calculate the twist boundary energy for the face-centred cubic (fcc) metals Al, Cu and Ni (ref. 44). Over a similar range of angles, the twist boundary energies obtained using APNGB model were shown to be in excellent agreement with those calculated using atomistic simulations^43^,^44^. Such a model is expected to be even more accurate for graphite, where the interlayer bonding (across the grain boundary) is very much weaker (and less stiff) compared with the intralayer bonding, and the dislocation cores are much wider than in the metals.

In the APNGB model, the system consists of a bicrystal; that is, two semi-infinite graphite crystals meeting at a common (0001) plane across which there is a rotation about the [0001] axis. The grain boundary energy consists of contributions from the elastic strain fields within the two semi-infinite crystals (elastic energy) and one associated with the bonding across the grain boundary plane (misfit energy). The strains in the crystals are calculated by minimizing the total energy with respect to the displacements (or disregistry) across the boundary plane. The misfit energy describes the energy change (relative to the perfect crystal) associated with disruption of the normal bonding across the basal plane between two crystals. See ref. 44 for more details.

The only input required for this calculation is the anisotropic elastic constants for graphite and the generalized stacking fault energy (GSFE)^49^. The anisotropic elastic constants are obtained from experimental measurements^50^; see Supplementary Table 3.

The GSFE *γ*_GSFE_ describes the variation of the energy of the system when the two semi-infinite crystals are rigid and uniformly displaced relative to one another in all directions parallel to the grain boundary plane (0001). Hence, the GSFE is a function of two displacement variables and, because of the bicrystal symmetry, is a periodic function. The GSFE can be represented by a function that respects the symmetry of the AB graphite crystals on the (0001) plane; in particular, we write^43^


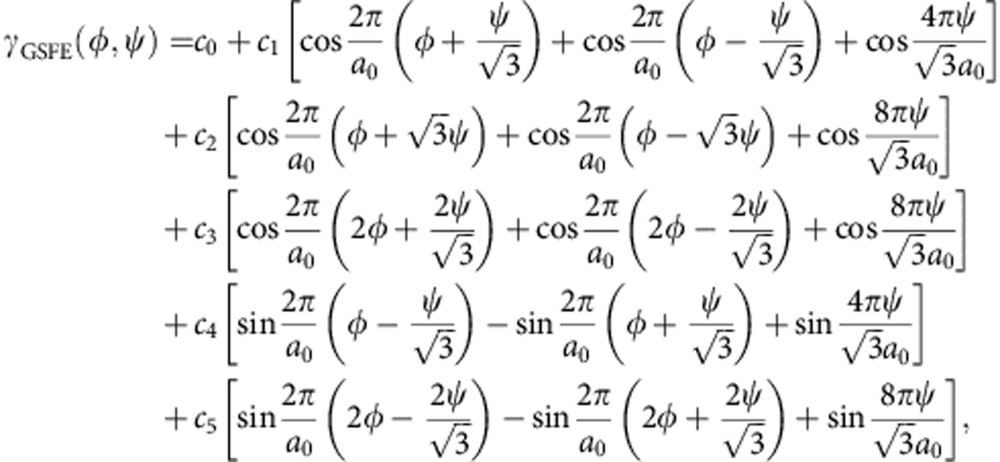


where *a*_0_ is the in-plane (0001) lattice constant of graphite, *φ* and *ψ* are the relative displacements between the upper crystal and the lower crystal, and *c*_0_=−3(*c*_1_+*c*_2_+*c*_3_). There are five independent parameters in this equation, that is, *c*_1_, *c*_2_, *c*_3_, *c*_4_ and *c*_5_. This function has been shown to accurately reproduce the GSFE in the (111) plane of several FCC metals^44^ and bilayer graphene.

The values of the parameters in the GSFE can be obtained by fitting to the shear elastic constant *C*_44_ across the (0001) plane in graphite (see above) and several energies in the GSFE landscape (Supplementary Fig. 2). We chose to fit to the stacking fault energy *γ*_sf_, the unstable stacking fault (saddle point) energy in the 
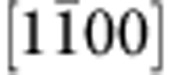
 (armchair) direction *γ*_sp_, the unstable stacking fault energy in 
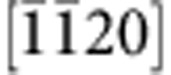
 (zig-zag) direction *γ*_sp'_, and the maximum energy in the GSFE landscape *γ*_peak_ as indicated in the predicted GSFE landscape shown in Supplementary Fig. 2.

We adopted the following strategy to obtain those values. In order to obtain *γ*_sp_, *γ*_sp′_, and *γ*_peak_, we rely on our recent ACFDT-RPA calculations on bilayer graphene to mimic the bonding across a (0001) plane in graphite. (ACFDT-RPA and QMC calculations provide the most accurate prediction of interlayer bonding in van der Waals materials compared with other first-principles methods.) While bilayer graphene energies are indeed different from the values for graphite, a recent critical examination^47^ of the differences between the binding energy and cleavage energy of graphite suggest that this approximation leads to an underestimate of the actual graphite interfacial energies of ∼15%. This is not surprising since the bond strength between van der Waals layers decays rapidly with interlayer separation. The obtained values are shown in Supplementary Table 4.

While we anticipate errors of order ∼15% in the values of *γ*_sp_, *γ*_sp′_ and *γ*_peak_ and of ∼100% in *γ*_sf_ (this error estimate is associated with the inability of LDA calculations to accurately model dispersion forces). Because *γ*_sf_ is so small, it plays a negligible role in determining the twist grain boundary energy and errors of this magnitude in *γ*_sf_ are unimportant. Overall, we expect the uncertainty in the GSFE surface to be, on average ∼15%.

## Additional information

**How to cite this article:** Wang, W. *et al.* Measurement of the cleavage energy of graphite. *Nat. Commun.* 6:7853 doi: 10.1038/ncomms8853 (2015).

## Supplementary Material

Supplementary Information Supplementary Figures 1-2, Supplementary Tables 1-4, Supplementary Note 1 and Supplementary References
