# Supplementary material for: Measurement of the cleavage energy of graphite
Source: Nat Commun. 2015 Aug 28;6:7853. doi: 10.1038/ncomms8853 (PMC4560750; doi:10.1038/ncomms8853)
Supplement: Supplementary Information — Supplementary Figures 1-2, Supplementary Tables 1-4, Supplementary Note 1 and Supplementary References [file ncomms8853-s1.pdf]

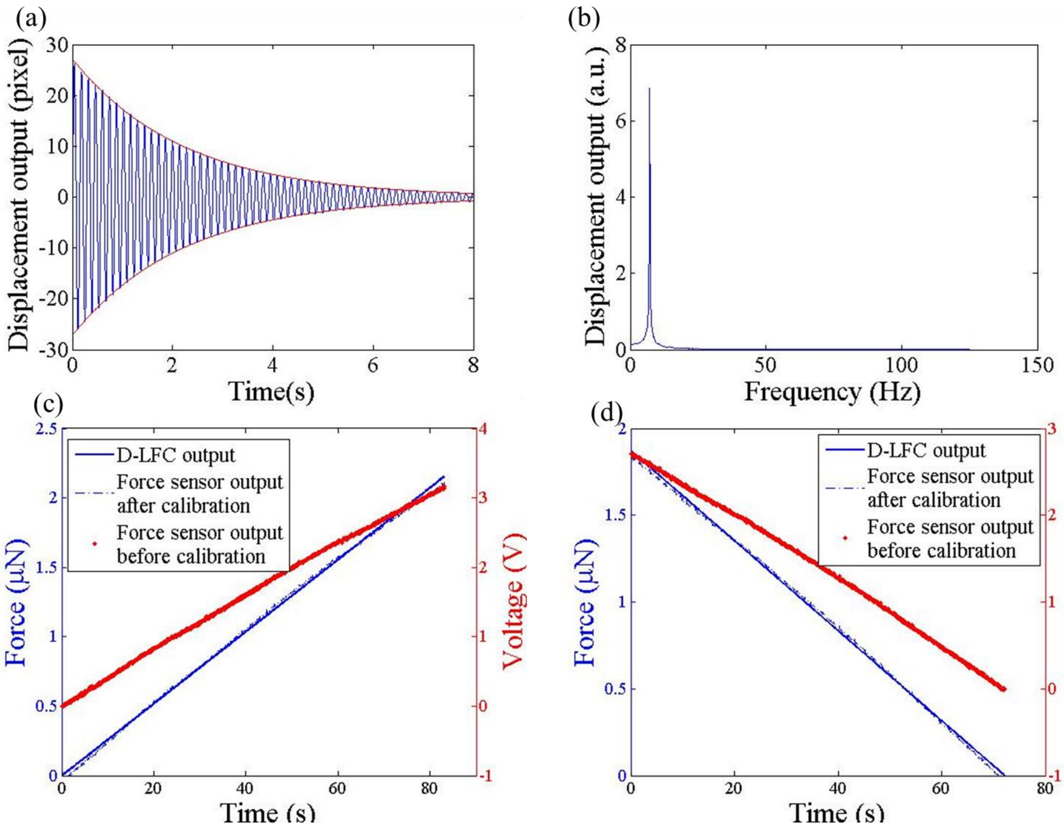

Supplementary Figure 1(a) The trajectory of the levitated pyrolytic graphite test sample (blue curve) and the envelope from free vibration theory (red curve). (b) The FFT of the displacement-time curve in (a) for pyrolytic graphite. (c) Loading calibration results for the force sensor. The blue line represents the D-LFC force output, the red dots represent the micro-force sensor voltage outputs and the blue dotted line represents the micro-force sensor force outputs after calibration. (d) Unloading calibration results for the force sensor.

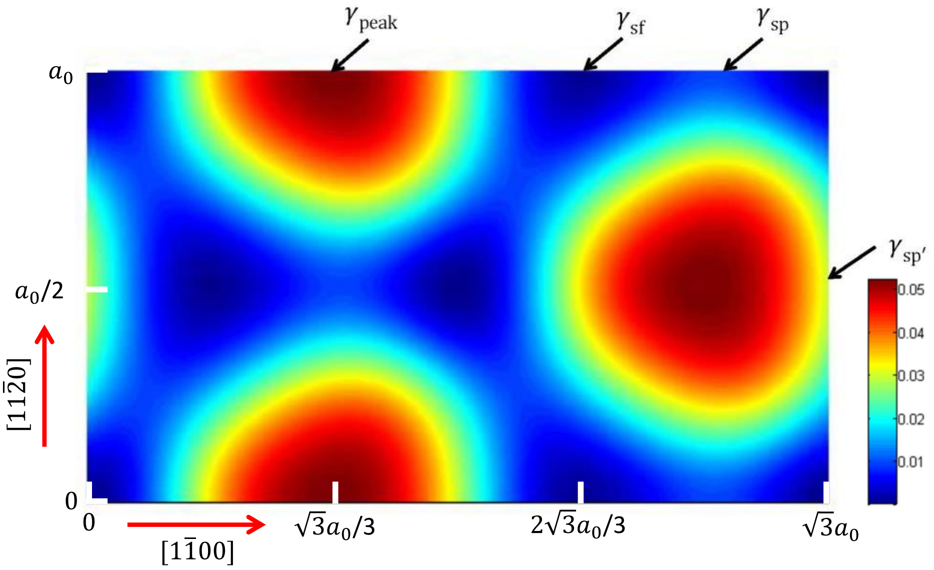

Supplementary Figure 2. The GSFE landscape in the graphite basal plane where the locations of the position of the points to which the GSFE is fitted are indicated. The colors indicate the magnitude of the GSFE as per the color bar on the right (J/m^2).

Supplementary Table 1. Reported experimental measurements of the graphite interlayer interaction energy (in J/m^2). The type of measurement is indicated as 2SE, EE, BE, and CE representing twice the surface energy, exfoliation energy, binding energy and cleavage energy, respectively. "Stack", where reported, indicates the layer stacking sequence.

Experiment method	Stack	Energy value	Type
Heat and wetting [1]	-	0.26	EE
TEM collapsed tube [2]	-	0.21 ± 0.06	BE
TD spectroscopy [3]	-	0.32 ± 0.03	EE
		0.37 ± 0.03	CE
CNT retraction [4]	Incommensurate	0.28 – 0.4	2SE
Mesoscopic [5]	AB	0.19 ± 0.01	BE
SEM peeling [6]		0.4 ± 0.18	2SE
		0.72 ± 0.32	2SE
This work	Incommensurate	0.37 ± 0.01	CE
	AB	0.39 ± 0.02	CE

Supplementary Table 2. Theoretical results for the graphite interlayer interaction energy (in J/m^2), where the notation is the same as in Supplementary Table 1.

Theory	Stack	Energy value	Type
DFT-LDA [7]	AB	0.12	BE
	AA	0.06	BE
DFT-LDA [8]	AB	0.31	BE
GGA [9]	AB	~0.03	BE
	AB	0.15	BE
vdW-DF [10]	AB	0.15	BE
vdW-DF [11]	AB	0.32	EE
vdW-DF [12]	AB	0.51	EE
vdW-DF [13]	AB	0.29	EE
	AB	0.31	CE
Semi-empirical [14]	AB	0.33-0.37	BE
Semi-empirical [15]	-	0.37	BE
QMC [16]	AB	0.34	BE
ACFDT-RPA [17]	AB	0.29	BE
LCAO-S2+vdW [18]	Turbostratic	0.43 ± 0.02	EE
	AB	0.49	EE

Supplementary Table 3. Elastic constants of graphite (GPa) [19].

C_{11}	C_{12}	C_{13}	C_{33}	C_{44}
**1060**	**180**	**7.9**	**36.5**	**5.05**

Supplementary Table 4. The energy values used in fitting the GSFE (mJ/m^2).

γ_{sf}	γ_{sp}	$\gamma_{sp'}$	γ_{peak}
**0.85**	**9.4**	**29.0**	**53.9**

Supplementary Note 1

Theoretical Predictions and Experimental Measurements of Interlayer Binding

There are four different types of energy measurements routinely used to describe the interlayer binding in layered materials such as graphite [20]; here we summarize the definitions of these and review the previous literature on graphite interlayer binding. The cleavage energy (CE) is the energy required to separate the material into two halves across a basal plane. The (0001) surface energy (SE) is the excess energy per unit area of free surface generated by cleavage; clearly CE is twice the SE. The exfoliation energy (EE) is the energy required to remove one (0001) atomic layer from the surface of the bulk material. Finally, the binding energy (BE) is the energy per layer per area required to separate the bulk into individual layers (usually by uniformly increasing the interlayer spacing in the entire crystal along the [0001] direction from its equilibrium value to infinity).

There have been several approaches to experimentally measuring the graphite interlayer interaction energy, often combining theoretical models and experimental measurements. The earliest experimental measurements were performed by Girifalco and Lad through a heat-of-wetting experiment; they reported an EE value of 0.26 J/m^2 (43 meV/atom) [1]. By analyzing TEM images of twisted collapsed nanotubes and using a force field to model its elastic properties, Benedict *et al.* extrapolated the BE to be 0.21 ± 0.06 J/m^2 (35 ± 10 meV/atom) [2]. Zacharia *et al.* [3] used thermal desorption spectroscopy to study the interaction of polychromatic hydrocarbons (PAHs) with the basal plane of graphite and found a larger EE value of 0.32 ± 0.03 J/m^2 (52 ± 5 meV/atom). They also estimated the CE of graphite to be 0.37 ± 0.03 J/m^2 (61 ± 5 meV/atom). Kis *et al.* measured the retraction force of carbon nanotube and reported the SE of a carbon nanotube (CNT) to be between 0.14 J/m^2 and 0.2 J/m^2 (22.9-32.7 meV/atom) [4]. Recently, by using an AFM to measure the profile of a graphite cantilever beam and combining FEM to calculate the strain energy, Liu *et al.* [5] reported the BE of graphite to be 0.19 ± 0.01 J/m^2 (31 ± 2 meV/atom); this value is, however, this value significantly underestimates the true value because of the use of the conventional L-J potential [20]. Michael *et al.* [6] used an AFM cantilever to measure the peeling force to separate

graphene from a multiwall carbon nanotube. By combining molecular mechanics simulations to calculate the contact area, they reported the SE to be 0.2 ± 0.09 J/m^2 (33 ± 15 meV/atom) or 0.36 ± 0.16 J/m^2 (59 ± 26 meV/atom), depending on the assumed conformation of the tube cross section. Supplementary Table 1 summarizes the experimental results for the graphite interlayer interaction energy.

From a theoretical standpoint, the challenge is to properly include both the-short-range chemical interactions/bonding within layers and the long range van der Waals interlayer interaction between layers. The former is associated with the overlap of electronic densities and the latter is associated with charge fluctuations (i.e., dispersion interactions/forces) [18]. Using the DFT-LDA method, Charlier *et al.* [7] found the BE of graphite to be 0.12 J/m^2 (20 meV/atom). Later Wang *et al.* [8] found the BE to be 0.31 J/m^2 (or 51 meV/atom) using a similar approach. The BE obtained using the GGA method was ~0.03 J/m^2 [9] which is even smaller than that reported based upon LDA. Researchers developed methods to account for the relatively weak long-range van der Waals interactions; these approaches include a semi-empirical method, a van der Waals density functional method (vdW-DF method), and quantum Monte Carlo (QMC) calculations. In the semi-empirical approach, the total energy is the sum of the DFT results and a van der Waals term (combined with a damping function). In the vdW-DF method, the total energy combines DFT results and a nonlocal term for the correlation energy described using an explicit analytical expression [21]. QMC is a many-body computational technique that can directly capture the dispersion force [22,23]. Rydberg *et al.* [9,10] calculated the BE using vdW-DF and reported 0.15 J/m^2 (or 25 meV/atom). Other applications of this approach yielded an EE in the 0.29-0.32 J/m^2 (or 47-52 meV/atom) [11-13] range. The BE found using the semi-empirical approach was between 0.33-0.37 J/m^2 (or 54-61 meV/atom) [14,15], which is larger than that of vdW-DF. The BE obtained by Spanu *et al.* [16] using electronic quantum Monte Carlo (QMC) methods was reported to be 0.34 J/m^2 (56 meV/atom). Lebègue *et al.* [17] reported the BE to be 0.29 J/m^2 (48 meV/atom) using the adiabatic-connection fluctuation-dissipation theorem in the random phase approximation (ACFDT-RPA). Savini *et al.* [18] applied DFT in the LDA and the LCAO-S^2 + vdW formalisms to find a BE of 0.43 ± 0.02 J/m^2 (70 ± 4 meV/atom) for a turbostratic graphitic stacking and

an EE of 0.49 J/m^2 (or 80 meV/atom) for the AB stacking. By applying the vdW-DF theory to calculate the adsorption of benzene and naphthalene on an infinite sheet of graphite, Chakarova-Käck *et al.* [13] predicted an EE of 0.29 J/m^2 (or 48 meV/atom) and estimated the CE to be 0.31 J/m^2 (or 50.5 meV/atom). The theoretical results for the graphite interlayer interaction energy are summarized in Supplementary Table 2.

Supplementary References

1 Girifalco, L. A. & Lad, R. A. Energy of cohesion, compressibility, and the potential energy functions of the graphite system. *J. Chem. Phys.* **25**, 693-697, (1956).

2 Benedict, L. X. *et al.* Microscopic determination of the interlayer binding energy in graphite. *Chem. Phys. Lett.* **286**, 490-496, (1998).

3 Zacharia, R., Ulbricht, H. & Hertel, T. Interlayer cohesive energy of graphite from thermal desorption of polyaromatic hydrocarbons. *Phys. Rev. B* **69**, (2004).

4 Kis, A., Jensen, K., Aloni, S., Mickelson, W. & Zettl, A. Interlayer Forces and Ultralow Sliding Friction in Multiwalled Carbon Nanotubes. *Phys. Rev. Lett.* **97**, (2006).

5 Liu, Z. *et al.* Interlayer binding energy of graphite: A mesoscopic determination from deformation. *Phys. Rev. B* **85**, (2012).

6 Roenbeck, M. R. *et al.* In Situ Scanning Electron Microscope Peeling To Quantify Surface Energy between Multiwalled Carbon Nanotubes and Graphene. *ACS nano* **8**, 124-138, (2014).

7 Charlier, J.-C., Gonze, X. & Michenaud, J.-P. Graphite interplanar bonding: electronic delocalization and van der Waals interaction. *Europhys. Lett.* **28**, 403, (1994).

8 Wang, Y., Scheerschmidt, K. & Gösele, U. Theoretical investigations of bond properties in graphite and graphitic silicon. *Phys. Rev. B* **61**, 12864, (2000).

9 Rydberg, H. *et al.* Hard numbers on soft matter. *Surf. Sci.* **532-535**, 606-610, (2003).

10 Rydberg, H. *et al.* Van der Waals Density Functional for Layered Structures. *Phys. Rev. Lett.* **91**, (2003).

11 Ziambaras, E., Kleis, J., Schröder, E. & Hyldgaard, P. Potassium intercalation in graphite: A van der Waals density-functional study. *Phys. Rev. B* **76**, (2007).

12 Ortmann, F., Bechstedt, F. & Schmidt, W. Semiempirical van der Waals correction to the density functional description of solids and molecular structures. *Phys. Rev. B* **73**, (2006).

13 Chakarova-Käck, S., Schröder, E., Lundqvist, B. & Langreth, D. Application of van der Waals Density Functional to an Extended System: Adsorption of Benzene and Naphthalene on Graphite. *Phys. Rev. Lett.* **96**, (2006).

14 Hasegawa, M. & Nishidate, K. Semiempirical approach to the energetics of interlayer binding in graphite. *Phys. Rev. B* **70**, (2004).

15 Hasegawa, M., Nishidate, K. & Iyetomi, H. Energetics of interlayer binding in graphite: The semiempirical approach revisited. *Phys. Rev. B* **76**, (2007).

16 Spanu, L., Sorella, S. & Galli, G. Nature and Strength of Interlayer Binding in Graphite. *Phys. Rev. Lett.* **103**, (2009).

17 Lebègue, S. *et al.* Cohesive Properties and Asymptotics of the Dispersion Interaction in Graphite by the Random Phase Approximation. *Phys. Rev. Lett.* **105**, (2010).

18 Savini, G. *et al.* Bending modes, elastic constants and mechanical stability of graphitic systems. *Carbon*

49, 62-69, (2011).

19 Cousins, C. & Heggie, M. Elasticity of carbon allotropes. III. Hexagonal graphite: Review of data, previous calculations, and a fit to a modified anharmonic Keating model. *Phys. Rev. B* **67**, 024109, (2003).

20 Gould, T. *et al.* Binding and interlayer force in the near-contact region of two graphite slabs: Experiment and theory. *J. Chem. Phys.* **139**, 224704, (2013).

21 Dion, M., Rydberg, H., Schröder, E., Langreth, D. C. & Lundqvist, B. I. Van der Waals Density Functional for General Geometries. *Phys. Rev. Lett.* **92**, (2004).

22 Drummond, N. & Needs, R. van der Waals Interactions between Thin Metallic Wires and Layers. *Phys. Rev. Lett.* **99**, (2007).

23 Sorella, S., Casula, M. & Rocca, D. Weak binding between two aromatic rings: feeling the van der Waals attraction by quantum Monte Carlo methods. *J. Chem. Phys.* **127**, 014105, (2007).
